# Simultaneous enzymatic saccharification and comminution for the valorization of lignocellulosic biomass toward natural products

**DOI:** 10.1186/s12896-018-0487-1

**Published:** 2018-12-12

**Authors:** Ronald R. Navarro, Yuichiro Otsuka, Masanobu Nojiri, Shigehiro Ishizuka, Masaya Nakamura, Kazuhiro Shikinaka, Kenji Matsuo, Kei Sasaki, Ken Sasaki, Kazuhide Kimbara, Yutaka Nakashimada, Junichi Kato

**Affiliations:** 1 Forestry and Forest Products Research Institute, National Research and Development Agency, Tsukuba, 305-8687 Japan; 2 Research Institute for Chemical Process Technology, National Institute of Advanced Industrial Science and Technology (AIST), Sendai, 983-8551 Japan; 3 Department of Molecular Biotechnology, Graduate School of Advanced Sciences of Matter, Hiroshima University, 1-3-1 Kagamiyama, Higashihiroshima, Hiroshima, 739-8530 Japan; 4 Department of Food, Agriculture and Bio-Recycling, Faculty of Engineering, Hiroshima Kokusai Gakuin University, 6-20-1 Nakano Aki-ku, Hiroshima, 739-0321 Japan; 5 Department of Applied Chemistry and Biochemical Engineering, Graduate School of Engineering, Shizuoka University, Naka-ku, Hamamatsu, 432-8561 Japan

**Keywords:** Lignocellulose, Saccharification, Comminution, Biorefinery, Cedar, Lignin

## Abstract

**Background:**

Large-scale processing of lignocellulosics for glucose production generally relies on high temperature and acidic or alkaline conditions. However, extreme conditions produce chemical contaminants that complicate downstream processing. A method that mainly rely on mechanical and enzymatic reaction completely averts such problem and generates unmodified lignin. Products from this process could find novel applications in the chemicals, feed and food industry. But a large-scale system suitable for this purpose is yet to be developed. In this study we applied simultaneous enzymatic saccharification and communition (SESC) for the pre-treatment of a representative lignocellulosic biomass, cedar softwood, under both laboratory and large-scale conditions.

**Results:**

Laboratory-scale comminution achieved a maximum saccharification efficiency of 80% at the optimum pH of 6. It was possible to recycle the supernatant to concentrate the glucose without affecting the efficiency. During the direct alcohol fermentation of SESC slurry, a high yield of ethanol was attained. The mild reaction conditions prevented the generation of undesired chemical inhibitors. Large-scale SESC treatment using a commercial beads mill system achieved a saccharification efficiency of 60% at an energy consumption of 50 MJ/kg biomass.

**Conclusion:**

SESC is very promising for the mild and clean processing of lignocellulose to generate glucose and unmodified lignin in a large scale. Economic feasibility is highly dependent on its potential to generate high value natural products for energy, specialty chemicals, feed and food application.

## Background

As petroleum reserves decline and it becomes more expensive, there is a need to identify and develop renewable and carbon-neutral alternatives [1–5]. Lignocellulosic biomass is an important resource for the production of fuel and chemicals, which is analogous to the role of crude oil in petrochemical refineries [6–9]. Ready supply, rapid renewability and carbon neutrality are among its salient features. The availability of non-food crops that grow in marginal or non-arable lands and the use of residues and wastes from agricultural crops further boost its potential. Conversion of holocellulose and the less exploited lignin components into high-value chemicals will enhance the profitability of biorefineries [10–12].

A number of treatment techniques using either biochemical or thermo-chemical methods have been employed for lignocellulosics processing [5, 13]. The ultimate goal is to expose and break cellulose from the lignin and hemicellulose complex [2, 6, 14–16]. So far, methods that rely on high temperature and acidic or alkaline conditions are the most reliable for large-scale operations [4, 9, 17]. Aggressive chemical reactions are necessary because of the strong interaction among the biopolymers [18]. However, extreme conditions generate toxic chemicals and destroy the native qualities of the products, particularly lignin. As an alternative, enzyme-based treatment has become a major focus of research [1]. Relative to chemical methods, it offers comparable yields without noxious side reactions due to the mild operating conditions. In order to maintain these vital qualities, an equally mild pre-treatment to render the biomass amenable to enzymatic hydrolysis is necessary. One candidate that has been tested in a pilot scale is the SPORL process [8, 9]. It utilizes dilute acid concentration to minimize the generation of toxic inhibitors during the alcohol fermentation process. The high conversion efficiency of 90% shows its potential for bioenergy generation.

Non-thermo-chemical based treatment that capitalizes on mechanical and enzymatic process has been considered in the past to completely avert the formation of undesirable contaminants [19]. Aside from the cleaner output that requires simpler downstream processing, it also generates unmodified lignin, which has a greater potential for the production of high-value materials. Products from this process could also find application in the feed and food sector. Currently, no reliable technology is capable of combined mechanical and biological process in a large scale. With this particular goal in mind, we have developed the “simultaneous enzymatic saccharification comminution” (SESC) process [12]. Its core is a readily scalable food-grade wet-milling equipment that utilizes fine zirconia beads to reduce particle size to less that 1 μm in conjunction with enzymes. In addition to exposing the cell wall and breaking down the lignocellulose complex at this level [12, 20] the grinding action increases the number of reactive sites and disrupts the crystalline structure of cellulose [14, 15, 21–23]. The susceptibility of cellulose to enzymatic hydrolysis is generally affected by the structure of the cellulosic fibers, and surface area and crystallinity are the most important structural features [16, 24]. Furthermore, the vigorous physical action can also destroy lignin-carbohydrate complexes to further enhance cellulose hydrolysis [20]. The SESC process is able to treat lignocellulosics at mild pH and temperature. The enzymatic saccharification being combined with comminution prevents major clogging of the comminution screen, keeps the reaction pressure well within the working conditions of the machine and lowers energy requirement for milling.

In this work, we further optimized the saccharification performance of SESC to increase product recovery. A large-scale operation that employs a commercially available beads milling system was also conducted to obtain basic data on wood processing capability and the resulting energy consumption. The commercial outlook based on its merits and limitations were closely examined and expounded.

## Methods

### Sample preparation

Chemicals. Deionized water, purified using a Milli-Q® Advantage A10® system (Millipore™, Eschborn, Germany), was used. Phosphate buffer was prepared from Na_2_HPO4 and NaH_2_PO4 purchased from Wako Pure Chemical Industries. The enzyme cocktail was prepared by combining equal amounts of commercial enzymes OPTIMASH XL containing cellulase and xylanase (10,300 U/g) and OPTIMASH BG containing xylanase and β-glucosidase (6200 U/g) from DuPont™ Genencor® Science. Jet milled cedar (18 μmΦ) was used for lab-scale and large milling operations.

### Laboratory-scale bead milling

Laboratory-scale SESC was conducted following our previous report (Shikinaka et al., 2016). To evaluate the effect of pH and the saccharification limit, a slightly modified procedure was employed. A mixture of 50 g of cedar powder (7.3% moisture content) and 440 g of 100 mM phosphate buffer in different pH conditions was subjected to bead milling (Starmill® LMZ015; Ashizawa Finetech Ltd., Japan) with zirconia beads (0.5 mmΦ). Enzyme cocktail at a loading of 0.2 mL/g wood biomass (corresponding to 30 FPU/g glucan) was added to the mixture. The peripheral bead velocity was set at 14.0 m/s. After 2 h of bead milling, the mixture obtained was subjected to overnight saccharification (first stage) with constant mixing at 50 °C. The saccharified slurry was then centrifuged at 10,000×*g* for 30 min. The recovered precipitate was combined again with a fresh batch of phosphate buffer and enzyme cocktail, and then saccharification (second stage) was allowed to proceed overnight at 50 °C. The generated product was centrifuged, and the collected precipitate was subjected to 2 h of second-stage bead milling with the same buffer and enzyme conditions but using a 0.1 mmΦ bead-size. The resulting slurry was once again subjected to overnight saccharification (third stage). After separating the precipitate, final saccharification (fourth stage) was performed using fresh enzyme and buffer. The supernatants generated from each saccharification stage were collected separately and subjected to high performance liquid chromatography (HPLC) analysis. The final lignin-rich precipitate was washed three times with an equal amount of ultrapure water with centrifugation at 10,000×*g* for 30 min.

In the experiments with two saccharification steps, the above procedure was followed, except that the extra saccharifications (i.e. the “second” and “fourth” steps described above) for each milling stage were omitted. Hence, the enzyme loading in this case was only half of the above experiments, which is similar to our previous condition [12].

During recycling experiments, two saccharification stages were employed. The supernatant from the first milling and saccharification stage was used during the second milling operation without any additional buffer or enzyme. In this case, the second batch of enzyme solution was added after the second milling, i.e., before the second saccharification step.

For ethanol fermentation, a non-buffered mixture of 50 g of cedar powder in 450 g pure water containing enzyme cocktail at 0.2 mL/g wood biomass was subjected to bead milling with zirconia beads (0.5 mmΦ) for 2 h. The peripheral bead velocity was set at 14.0 m/s. After the first milling, the slurry was recovered and then subjected to overnight saccharification at 50 °C. The saccharified slurry was subjected to a second milling for 2 h with an additional enzyme at similar amount and using smaller zirconia beads (0.1 mmΦ). After the second saccharification, the slurry was subjected to alcohol fermentation by adding one pack of commercial dry yeast (Kyoritsu Foods Co., Ltd., Tokyo, Japan). Samples were taken at specific time intervals and analyzed for glucose and ethanol. The fermentation was terminated when the glucose and ethanol concentrations stabilized.

### Large-scale beads milling

**Fig. 1:**
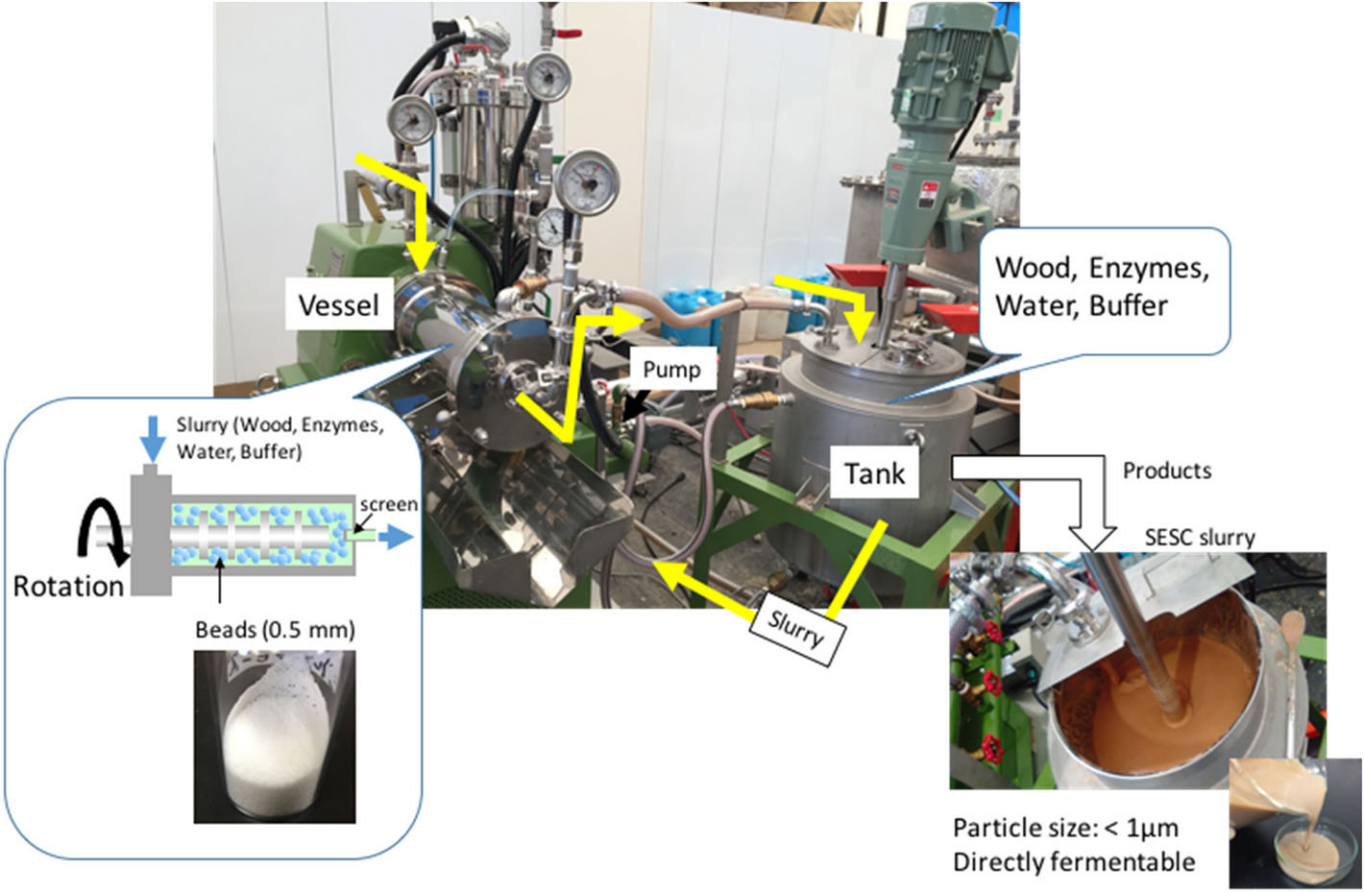
Large-scale SESC system. The vessel contains 0.5-mm zirconia particles for comminution. Ten liters of 10% aqueous slurry (wood powder, enzyme and buffer) recirculate continuously from the tank through the vessel. The vessel contents are subjected to rotational grinding action and the centrifugal force generated facilitates bead separation by a screen near the outlet. Around 3-h of treatment is necessary to achieve a particle size of < 1 μm

Large-scale SESC was performed using a Starmill LME4 bead mill unit (Ashizawa Finetech Co., Ltd.) (Fig. 1). The milling vessel contained 0.5-mm zirconia beads. The wood slurry recirculates through this vessel, where the contents are subjected to rotational grinding. The resulting centrifugal force facilitates bead separation and at the same time prevents clogging of the comminution screen. Slurry preparation was carried out by combining 1 kg jet milled cedar with 100 mL of 1 M phosphate buffer (pH 7), enzyme cocktail (0.2 mL/g wood biomass), and close to 10 L distilled and deionized water. The use of pH 7 buffer stock solution in this case results in the maintenance of around pH 6 during milling. The milling speed was set at 14.5 m/s and the slurry circulation flow rate was 2 L/min. The chamber temperature was maintained at 50 °C by a chiller unit. Samples were taken at specific time intervals and then quickly analyzed with a SALD-2300 laser diffraction-type particle size analyzer (Shimadzu Corporation). When the median particle size had dropped to 1 μm, the operation was terminated. Additional saccharification was carried out at 55 °C for 24 h with a fresh batch of enzyme cocktail at similar loading.

### Analytical methods

HPLC was used for simultaneous sugar and alcohol analysis. The slurry samples were first centrifuged, and the supernatants obtained were filtered through a syringe filter with a nylon membrane (porosity 0.2 mm) prior to injection into the HPLC column. Diluted samples were analyzed by HPLC (LC-20 AD, Shimadzu Co. Ltd. Japan) on a combined ligand exchange and size exclusion chromatography column (SUGAR SP0810, 7 μm column, 8.0 × 300 mm; Shodex Co. Ltd. Japan) at 80 °C with water flowing at 1 mL/min. The eluting sugar compounds and ethanol present in the sample were detected using a refractive index (RI) detector (RI-201H, Shodex Co. Ltd. Japan) and identified by comparing their retention times with those of purified standards.

Furfural and hydroxymethyl furfural analyses were carried out in a Jasco Gulliver Series HPLC (Jasco Corporation, Tokyo, Japan) equipped with a U*V*/*v*is detector (UV970, JASCO Corp., Japan) set at 280 nm and a 4.6 × 250 mm Inertsil ODS-3 column (GL Sciences Inc., Tokyo, Japan). Temperature was maintained at 40 °C by a CO 1565 Jasco column oven. During analysis, exactly 20 μL of sample was injected and eluted at a flow rate of 1.0 mL/min of solvent A (10 mM H_3_PO_4_) with a linear gradient from 10 to 50% of solvent B (acetonitrile).

Total organic carbon (TOC) and total nitrogen (TN) values were analyzed with a TOC-TN analyzer (TOC-L/TNM-L; Shimadzu, Kyoto, Japan). Nitrogen analysis of dried lignin-rich precipitate was performed by the dry combustion method (Vario MAX CN, Elemental, Langenselbold, Germany). All chemical analyses were conducted in duplicate, and average values are reported. The particle size distribution of milled wood samples was measured using a SALD-2300 laser diffraction particle size analyzer.

## Results

### Saccharification efficiency and enzyme loading as a function of pH

Using untreated cedar as a representative softwood, we previously reported the ability of SESC to deliver a saccharification efficiency that is comparable to common thermo-chemical methods [12]. Around 70% sugar recovery based on the total cellulose content was achieved near the pH optimum of cellulase enzyme (pH 4.8) [25]. However, the saccharification of lignocellulose is not necessarily highest at this condition but at pH 5.5–6 [19, 26, 27] due to the less occurrence of non-productive enzyme adsorption to lignin at higher pH [26–29]. Molecular dynamics simulation of model lignocellulosic biomass has confirmed that lignin binds both to the enzyme-binding sections of cellulose, as well as to cellulose-binding sections of the enzyme [30]. The interaction is mainly governed by electrostatic forces, so that by increasing pH beyond the isoelectric point (pI) of cellulase (pH 4.5–5), repulsions between the negatively charged enzyme and the lignin molecules are enhanced [26]. However, previous evaluations have utilized lignin whose native structure has drastically changed during chemical recovery. Now, a fairly recent report has suggested the possibly low affinity of enzymes to lignin in its natural form, [31] which is considered to be the state of the lignin from SESC. Hence, in the present study, basic experiments were conducted to determine how the SESC lignin affects cellulose saccharification at different pHs.

**Fig. 2:**
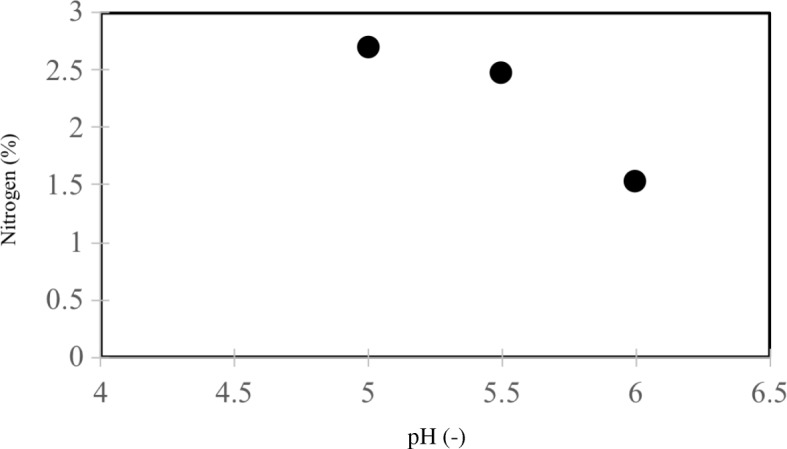
Nitrogen analysis of dried lignin-rich precipitate generated from the SESC treatment of cedar at different pH values. The nitrogen content represents the amount of enzyme in the residue. Values are averages of two analysis runs

Saccharification of SESC-treated cedar powder was performed using a higher enzyme loading in order to establish the limit of sugar recovery independent of the amount of enzyme. Furthermore, a second stage of milling utilizing smaller zirconia beads was also conducted to expose as much lignin. The results confirmed that saccharification by SESC increases at higher pH (Table 1). Total sugar recovery, in terms of released glucose and cellobiose relative to the total cellulose, improved to 80% above pH 5.5. At pH 6, the recovery was 83%, which is 20% higher than the previous result of 70% at pH 4.8. This finding clearly conforms to the established notion regarding the loss of enzymatic activity from non-productive lignin adsorption. This was supported by TN and elemental analyses (particularly N) of both the saccharification supernatant and the dried precipitate, respectively. (The nitrogen content of control cedar powder was not detected; thus the N content can be used to approximate the amount of enzyme present.) At pH 6, TN analysis showed that 25% of the added enzyme remained free during the first saccharification stage, which is the period when the highest number of binding sites on lignin are available. At pH 5 and 5.5, these values were only 8.5 and 7%, respectively. A more direct verification of enzyme-lignin interaction was also obtained from the dried solid fraction, which exhibited a decreasing nitrogen (i.e. enzyme) content at increasing pH (Fig. 2). To some extent, the minimal difference of the TN values at pH 5 and 5.5 does not appear to account for the relatively large discrepancy of their sugar recoveries. In addition, the similar saccharification performances at pH 5.5 and 6 do not correspond well with their TN difference. To reconcile these results, it must be emphasized that an excess amount of enzyme was used in these runs, so that despite the higher occurrence of non-productive enzyme binding (particularly at the lower pH), the greater enzyme loading overcame this limitation. This finding was validated during subsequent experiments with half the amount of enzyme using two saccharification stages, wherein a greater difference in sugar recoveries at pH 5.5 and 6 was apparent. At this condition, the sugar recovery at pH 6 (81%) was distinctly higher than that at pH 5.5 (71%).

**Table 1:** Percentage extraction of sugars from SESC-treated cedar slurry at different pH values in different saccharification stages (1st, 2nd, 3rd and 4th)

pH (−)	Glucose extraction (%)					Cellobiose extraction (%)					Total sugar
	1st	2nd	3rd	4th	Total	1st	2nd	3rd	4th	Total	
5	33.6	16.7	6.3	1.2	57.8	10.7	0	0	0	10.7	67.3
5.5	35.5	27.7	10.5	2.9	76.6	3.2	1.2	0	0	4.4	81.0
6	41.6	21.6	10.0	3.5	76.7	6.0	1.1	0	0	7.1	83.8
6.5	12.8	34.7	14.8	4.8	67.1	8.1	5.7	0	0	13.8	80.9
7	3.3	21.5	14.9	11.0	50.7	6.6	12.0	10.5	1.1	30.2	80.9

Jet-milled slurry was subjected to two-stage bead milling at 50 °C using 0.5- and 0.1-mmΦ zirconia beads, respectively. An enzyme cocktail of 0.2 mL/g wood biomass (30 FPU/g glucan) was added in each saccharification stage (see Experimental Methods). Phosphate buffer (100 mM) was used for pH control. All values are the average of two analysis runs

### SESC supernatant recycling

Enzyme cost may also be reduced through recycling [32]. Aside from reusing the buffer solution and enzyme, this also concentrates the product, thus making recovery operations more practical. Indeed, one limitation of wet-type milling is sample dilution, which lowers productivity by increasing water and energy consumption. However, solution recycling also concentrates other chemicals that may inhibit enzymatic activity [13, 32]. It may also initiate product inhibition. Hence, we compared the saccharification efficiencies of recycled and non-recycled systems operating at pH 6 (established above as the optimum pH). For this test, instead of using a fresh batch of buffer and enzyme during the second milling stage, the supernatant from the first milling was directly used. Because of the presence of the carry-over enzymes from the first stage, the second batch of enzyme was added after and not during the second milling. This procedure was found to generate a supernatant with at least 70% higher glucose concentration than the non-recycled system. This increase was achieved without a negative impact on saccharification efficiency. Total sugar recovery in the recycled system was comparable to the above value of 81% for a two-stage saccharification experiment at pH 6 (Table 2), which indicates no apparent inhibition of enzymes. On the contrary, supernatant re-use even channeled the reaction toward the complete conversion of cellobiose to glucose. One way to interpret this finding is that the higher enzyme concentration due to the carry-over from the first saccharification may have enhanced the overall β-glucosidase activity. Furthermore, the addition of fresh enzyme after milling during the recycle run may have resulted to higher enzymatic activity due to the absence of shear stress from beads contact. However, the extent of this effect, which could further contribute towards improving the process efficiency, is still under detailed evaluation.

**Table 2:** Percentage extraction of sugars from SESC-treated cedar slurry with different numbers of saccharification stages (four and two) with and without supernatant recycling

Number of saccharifications	Glucose extraction (%)					Cellobiose extraction (%)					Total sugar
	1st	2nd	3rd	4th	Total	1st	2nd	3rd	4th	Total	
4	41.6	21.6	10.0	3.5	73.2	6.0	1.1	0	0	7.1	83.8
2	26.9	44.1	–	–	71.0	6.6	3.4	–	–	10.0	81.0
2 (recycle)	–	80.0	–	–	80.0	–	0	–	–	0	80.0

Jet-milled slurry was subjected to two-stage bead milling at 50 °C using 0.5- and 0.1-mmΦ zirconia beads, respectively. An enzyme cocktail of 0.2 mL/g wood biomass (30 FPU/g glucan) was added in each saccharification stage (see Experimental Methods). Phosphate buffer (100 mM) was used to maintain pH 6. All values are the average of two analysis runs

### Alcohol fermentation of SESC-treated cedar slurry

**Fig. 3:**
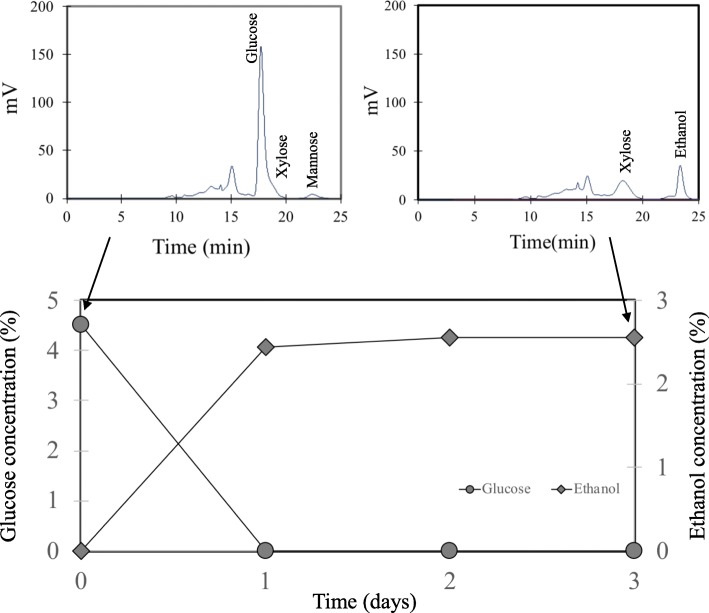
Time course of glucose and ethanol concentration during the fermentation of SESC-treated cedar slurry. The slurry, which had undergone two stages of milling and saccharification, was directly subjected to alcohol fermentation. The high percentage conversion of ethanol based on the final ethanol concentration and the initial glucose concentration from the saccharification stage (109.8% corresponding to 0.57 g ethanol/g glucose) suggests the generation of additional glucose during the fermentation process. Values are averages of two analysis runs

**Fig. 4:**
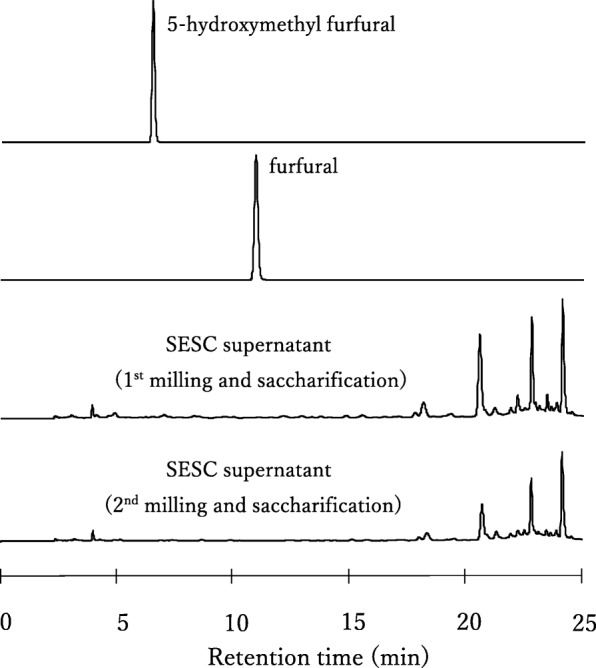
HPLC spectra of representative SESC slurry supernatants from the first and second milling and saccharification stages. The standard furfural and hydroxyl methyl furfural (HMF) concentrations are both 10 mg L^− 1^, which is 50 times lower than their inhibition concentrations for alcohol fermentation. Note the absence of furfural and HMF peaks in both SESC samples

Biomass has the potential to generate fermentation inhibitors particularly furfural, weak acids and phenolic compounds during chemical treatment at high temperature [33] These substances disrupt cellular replication, sugar metabolism and the membrane integrity of fermentative microorganisms. In our previous work, this was not a major concern since a high conversion of glucose to alcohol during fermentation of sugar-rich supernatant from SESC was achieved [12]. In the current evaluation, we went a step further by directly fermenting the slurry. This would test any adverse contribution from the non-degraded lignin-rich precipitate. Fermenting the slurry also sought to improve the overall saccharification through additional cellulose hydrolysis during fermentation. Results showed that even under this situation, fermentation achieved a high ethanol yield (Fig. 3). HPLC analysis did not detect furfural and hydroxylmethyl furfural from the samples (Fig. 4). We were able to achieve 76% sugar recovery, which is slightly higher than the saccharification at similar pH (70%). The continuous rise of ethanol after the first day (Fig. 3), despite the non-detection of glucose at this time, could represent brief episodes of simultaneous saccharification and fermentation (SSF). Indeed, the yield of ethanol that exceeded the conversion of available glucose from the saccharification stage (109.8% corresponding to 0.57 g ethanol/g glucose) suggested that additional glucose may have been generated during the ethanol fermentation stage. This may result from the declining product inhibition from glucose at this stage, thus increasing the extent of hydrolysis particularly of residual cellulose and sugar oligomers [34]. We also considered the possible decrease in non-productive enzyme binding due to ethanol interaction with lignin according to a recent work describing the positive effect of ethanol organosolv lignins on the enzymatic hydrolysis of pure cellulose [35]. Modification of lignin through ethanol organosolv processes albeit at high temperature may reduce non-productive enzyme binding. However, we did not detect any correlation between ethanol concentration (up to 10%) and saccharification efficiency of the wood slurry at ambient temperature (data not shown).

### Energy requirement for large-scale SESC

**Fig. 5:**
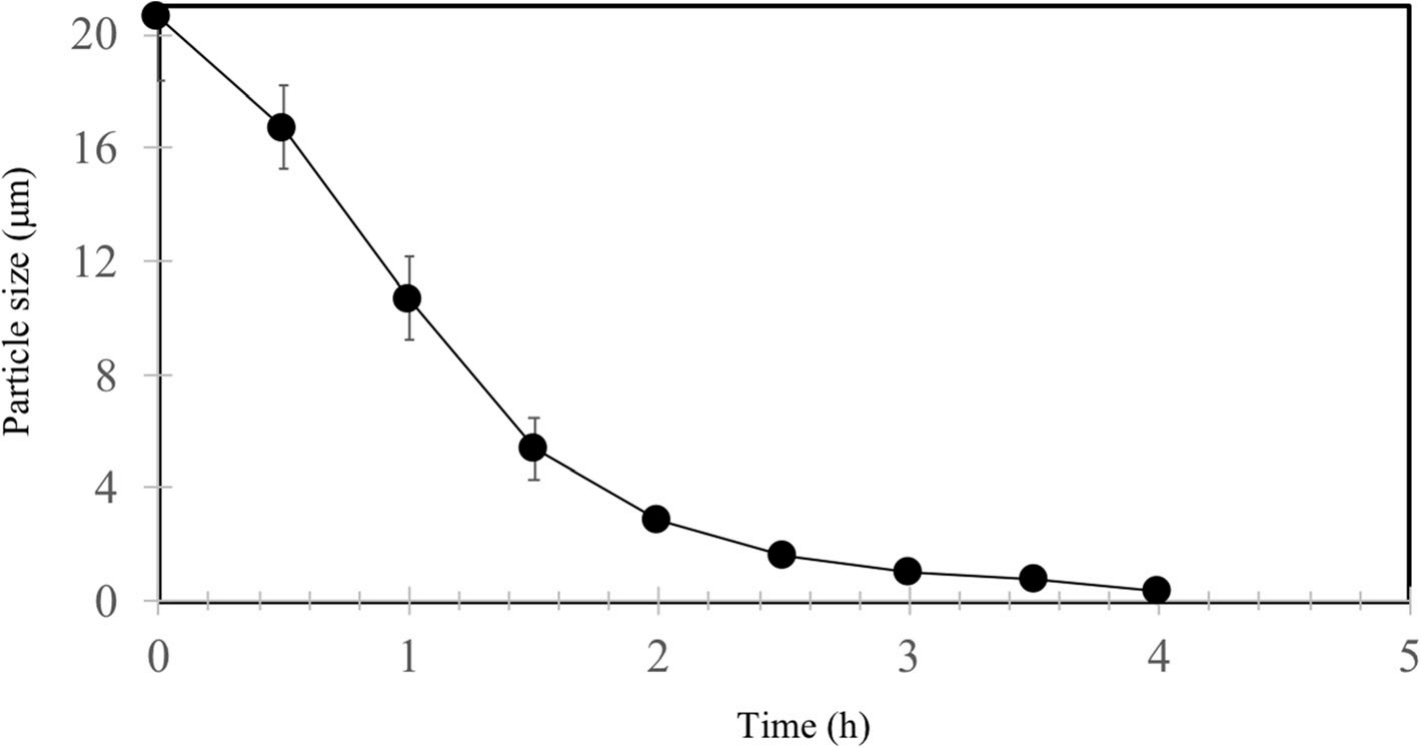
Time course of particle size during beads milling of 10-L slurry of jet-milled cedar using 0.5 mm beads size. Values are averages of triplicate runs

The large-scale physical comminution of 10-L jet-milled cedar slurry (10% aqueous mixture) required 3 h to achieve the target median particle size (< 1 μm) (Fig. 5). Sugar analysis of the supernatant revealed a saccharification efficiency of 60% for a single-stage SESC operation. This performance is comparable to thermo-chemical methods for softwood biomass treatment (ranging from 60 to 80%) [36]. The wet milling equipment, which employed a 5.5-kW motor operating a little below its capacity (5 kW) for 3 h, registered an average energy consumption of 50 MJ/kg biomass. It must be mentioned that this value is solely for bead comminution. Preliminary size reduction to achieve the appropriate size for bead milling also requires additional energy.

## Discussion

Mechanical comminution coupled with enzymatic reaction is the eco-friendliest approach for lignocellulosic processing. However, due to the negative views on the use of enzymes and intensive energy, measures must be undertaken to compensate for these limitations. First, it is essential to maximize the enzymatic activity through the optimization of reaction conditions. The results from the pH evaluation during SESC treatment confirmed that better saccharification is achieved at pH 6 due to reduced non-productive enzyme binding. However, quantification of free enzymes indicated the difficulty of completely eliminating this phenomenon even at the optimum pH. The operation protocol may also be modified to maximize enzyme productivity. We have confirmed the feasibility of enzyme recycling during SESC with a favorable effect on sugar recovery. The resulting concentrated product also makes downstream processing more practical. Finally, in-house enzyme production using substrates from lignocellulosic processing, such as nutrient-rich waste streams, could also help address the economic shortcomings of enzyme-based pre-treatment [19] by reducing purification, concentration, storage, and shipment expenses that are generally incorporated in the price of commercial enzymes [13]. The uncontaminated and natural characteristic of any SESC-generated waste is well adapted for this strategy.

SESC optimization showed that an enzyme concentration of 0.2 mL/g wood biomass (corresponding to 30 FPU/g glucan above) for a single stage milling is necessary to achieve around 60% saccharification efficiency. On the other hand, established methods such as the SPORL process, which combines mild thermo-chemical treatment with enzymatic reaction has been reported to achieve a higher efficiency (90%) at lower enzyme loading (15 FPU). This is to be expected considering the possible synergism between acid and enzymatic hydrolysis. However, the mild operating conditions of SESC eliminates energy requirement for thermo-chemical reaction and at the same time generate less unwanted chemical by-products including furfural derivatives. In fact, the complete absence of inhibitors allowed for the direct alcohol fermentability of the sugar-rich SESC slurry at high sugar to ethanol conversion.

To address the intensive energy requirement of SESC, energy usage generally declines at higher operation scale. For example, a lab-scale wet planetary ball milling requires at least 2000 MJ/kg biomass (for rice straw), but an industrial system operating at 20 tons of biomass per h with a 400-kW engine only consumes 0.64 MJ/kg for the same level of treatment [15]. This provides a positive energy forecast for large-scale processing, but actual evaluations are necessary for SESC. Another path to economic feasibility would be to utilize alternative energy sources such as watermills or windmills. Unlike thermo-chemical methods, beads milling mainly requires a continuous rotary motion, so that it may be configured to work from these natural sources. However, due to their maximum power output limitations (3–10 HP) [37], an assembly of milling units functioning at the operation scale threshold must be considered.

It is also important to point out that the hardware for the commercialization of SESC are already available. Milling systems of various configurations and capacities are currently being employed for pharmaceutical, special chemicals and even food manufacturing. A grinding and dispersing machine (LME3000, Ashizawa Finetech) equipped with a 220-kW motor can be used to process 3 tons of wood slurry in a single operation. For much larger output, ultrafine grinders used in the mining industry, such as ISAMill™ with motors having power ratings up to 8000 kW and which operates under the same principle of rotating media comminution are suitable candidates [38]. These enormous milling machines may drive biorefineries of the future.

**Fig. 6:**
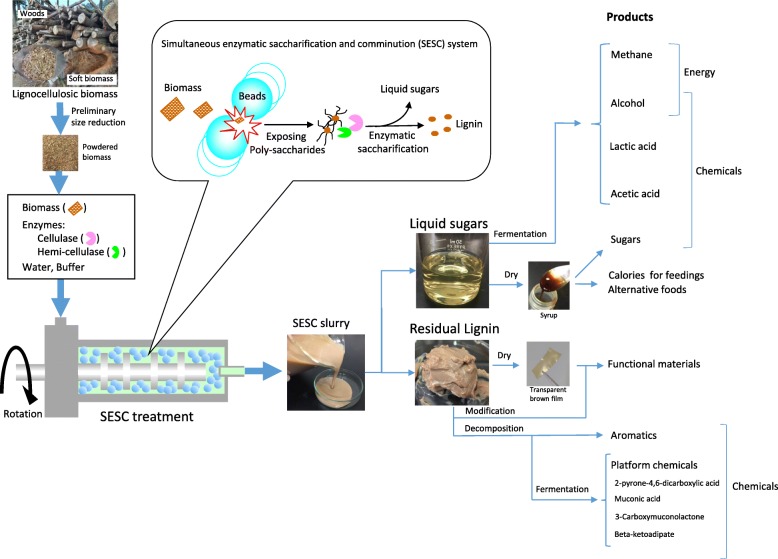
Valorization of lignocellulosic biomass by the SESC process. The major products, consisting of sugars and unmodified lignin, may be processed to generate energy, specialty chemicals, materials and even food-related compounds

The commercial viability of a biorefinery could best be elevated through product diversification. Lignocellulosics utilization should not only be limited to biofuels but instead, high-value chemicals must be prioritized [39]. This path to offset costs is highly suited for SESC. Figure 6 provides a list of products that may be directly and indirectly produced through this process. The mild nature of mechanical comminution and the specificity of the enzymatic reactions means that the major output will be a sugar-rich supernatant and a native lignin-rich precipitate. The glucose-rich SESC supernatant and even the slurry itself can be directly used as a fermentation substrate to produce fuel compounds such as ethanol and methane as well as other important chemicals like lactic acid and acetic acid. Though not covered in this work, SESC also generated significant amounts of xylose, which is another high-value product (Fig. 3).

Non-degraded lignin is an important component that may brighten the financial outlook of SESC. Currently, majority of lignin are derived from the kraft process of wood pulping. Kraft lignin represents 90% of all lignin produced, but the presence of aliphatic thiol groups gives the material an unpleasant odor, thus confining its use to in-house fuel [40]. The availability of high-quality sulfur-free lignin in large quantities could create novel applications. For instance, we have used the lignin-rich SESC precipitate to synthesize composite materials with high fraction strain, elasticity and fire resistance, thus making the biopolymer an ideal antiplasticizer or thermoplastic elastomer [12]. Non-deteriorated lignin was also found to impart excellent heatproof property to synthetic polymers such as poly(ethylene carbonate) [41]. Lignin may also be decomposed to generate aromatic building blocks for various materials. Generally, lignin obtained through thermo-chemical reactions has undergone extensive modification so that they have fewer β-O-4 linkages. This lignin is highly condensed due to the formation of strong and recalcitrant carbon-carbon bonds [42], making the polymer less susceptible to complete depolymerization by chemical or microbiological means. In this connection, we have previously shown that SESC-lignin is comparable to neat lignin in terms of the aromatic yield following nitrobenzene oxidation, a clear indication of its non-denatured characteristics [12]. Easier liberation and higher yields of aromatics during depolymerization is essential for the production of various platform chemicals. One of the most significant lignin-derivatives that we have developed is 2-pyrone-4,6-dicarboxylic acid (PDC), which can be employed as an epoxy adhesive [43], flocculant for radioactive Ce trapping [44] as well as a building block for biobased polymers such as polyamide, polyester and polyurethane [45–49]. Lignin may also be used as a raw precursor for other compounds, such as DMSO, vanilla, phenol, and ethylene [39]. Different types of biopolymers may also be produced from other lignin-derived platform chemicals, such as muconate, muconolactone and β-ketoadipate [50–53].

Future trends in lignocellulosic processing could also be directed for feed or even food manufacturing [54]. Feed production alone enhances food security by decreasing the fraction of arable land that must be used to grow crops for animal use [55]. In this connection, wood molasses has long been used to supplement animal feeds [56]. As an additive to swine, cattle or poultry feeds, its nutritional value is comparable to molasses from other sources such as beet and sugarcane [54]. However, thermo-chemical pre-treatment complicates purification and lowers the quality and stability of wood molasses [57]. In contrast, the uncontaminated and natural SESC extract would only require a simple evaporation step to generate a molasses-like syrup (Fig. 6). In addition to sugar, sulfur-free SESC lignin could also be utilized as animal feed supplement. Pure lignin and its derivatives provide health benefits to monogastric animals [58].

Food-related uses of wood are currently limited. Wood cellulose may serve as thickener or binding agent in food products [59] or may be included in dietary supplements for fat adsorption [60]. The utilization of wood for its caloric value, however, is yet to be realized. By employing food-grade buffer and enzymes, SESC sugar concentrate can be made edible for this function. This applies to other fermentation products including ethanol and acetic acid. In fact, our laboratory is currently developing a spirit liquor from SESC-treated wood. As a strong indication of the mildness of reaction, the distilled alcohol has the aroma of the particular tree species employed. Previously, a novel procedure that converts lignocellulosic cellulose into starch was reported; it employs a cocktail of enzymes that direct the transformation of cellulose into amylose [11]. Despite the initially limited production capacity and economic hurdles, the researchers were very confident of its potential in addressing future food shortages. We have a similar outlook for the SESC process. Though more detailed and strict tests are necessary to realize this goal, ‘wood for food’ research is where SESC-based lignocellulosic valorization can create a unique and vital niche.

## Conclusion

The simultaneous enzymatic saccharification and comminution (SESC) process was recently developed to create a mild and environmentally-friendly process for the treatment of lignocellulosic biomass. Process optimization and commercial feasibility evaluation were conducted to further enhance its performance in generating natural products that may be used for a wide variety of novel applications. The pH has a significant effect on the saccharification efficiency of SESC, reaching 80% at pH 6. Solution recycling resulted in higher product concentration without affecting overall sugar recovery. The direct alcohol fermentation of SESC-generated slurry allows for the complete conversion of glucose to ethanol. SESC has huge potential to generate energy and high value products from woody biomass for industrial, agricultural and even feed and food applications, thus possibly countering major concerns regarding its enzyme and energy requirements.

## Abbreviations

HPLC: High performance liquid chromatography
PDC: 2-pyrone-4,6-dicarboxylic acid
SESC: Simultaneous enzymatic saccharification and comminution
TN: Total nitrogen
TOC: Total organic carbon
